# Supplementary material for: Migration and its impact on universal HIV testing and treatment in the HPTN 071 (PopART) study communities
Source: PLOS Glob Public Health. 2026 Jun 1;6(6):e0005357. doi: 10.1371/journal.pgph.0005357 (PMC13225650; doi:10.1371/journal.pgph.0005357)
Supplement: S1 File — (DOCX) [file pgph.0005357.s001.docx]

### Supplementary material S1 – Population cohort characteristics and rate of out-migration

| **Variable** | **Category** | **Distribution at entry, N (%)** | **Migrated out during follow up period, N** | **Follow up time (person years)** | **Crude rate of out-migration per 100 person-years (95% CI)** | **Adjusted† Rate Ratio (95% CI)** | **p-value** |
| --- | --- | --- | --- | --- | --- | --- | --- |
| **Zambia** | | | | | | | |
| **Gender¥ (6 MV)** | **Men** | 7,260 (27.3) | 2,054 | 16,376 | 12.5 (12.0 - 13.1) | 1 (reference) | <0.001 |
|  | **Women** | 19,311 (72.7) | 4,425 | 44,580 | 9.9 (9.6 - 10.2) | 0.80 (0.75-0.86) |  |
| **Age group¥ (7 MV)** | **18-19** | 3,953 (14.9) | 1,152 | 8,848 | 13.0 (12.3 – 13.8) | 0.81 (0.70-0.93) | <0.001 |
|  | **20-24** | 7,997 (30.1) | 2,397 | 17,640 | 13.6 (13.1 - 14.1) | 1 (reference) |  |
|  | **25-29** | 5,555 (20.9) | 1,418 | 12,542 | 11.3 (10.7 – 11.9) | 0.87 (0.81-0.93) |  |
|  | **30-34** | 4,122 (15.5) | 823 | 9,797 | 8.4 (7.8 - 9.0) | 0.67 (0.61-0.73) |  |
|  | **35-39** | 2,906 (10.9) | 427 | 7,229 | 5.9 (5.4 – 9.0) | 0.48 (0.43-0.53) |  |
|  | **40-44** | 2,037 (7.7) | 262 | 4,899 | 5.3 (4.7 - 6.5) | 0.41 (0.36-0.46) |  |
| **HIV status at entry (642 MV)** | **HIV-** | 20,555 (79.3) | 5,119 | 47,000 | 10.9 (10.6 - 11.2) | 1 (reference) | <0.001 |
|  | **HIV+ (SR-)** | 2,318 (8.9) | 648 | 5,129 | 12.6 (11.7 - 13.6) | 1.28 (1.17-1.39) |  |
|  | **HIV+ (SR+, ART-)** | 810 (3.1) | 180 | 2,107 | 8.5 (7.4 - 9.9) | 0.99 (0.85-1.16) |  |
|  | **HIV+ (SR+, ART+)** | 2,252 (8.7) | 365 | 5,237 | 7.0 (6.3 - 7.7) | 0.95 (0.85-1.07) |  |
| **Socio-economic**  **quintile**  **(268 MV)** | **1 (Poorest)** | 5,812 (22.1) | 1,572 | 13,543 | 11.6 (11.0-12.2) | 1 (reference) | <0.001 |
|  | **2** | 4,537 (17.2) | 1,107 | 10,235 | 10.8 (10.2-11.5) | 0.85 (0.78-0.93) |  |
|  | **3** | 5,838 (22.2) | 1,362 | 12,666 | 10.8 (10-2-11.3) | 0.77 (0.70-0.84) |  |
|  | **4** | 5,159 (19.6) | 1,170 | 12,236 | 9.6 (9.0-10.1) | 0.63 (0.57-0.69) |  |
|  | **5 (Richest)** | 4,963 (18.9) | 1,193 | 11,663 | 10.2 (9.7-10.8) | 0.69 (0.62-0.76) |  |
| **Education**  **(122 MV)** | **None** | 478 (1.8) | 113 | 1,118 | 10.1 (8.4-12.2) | 1.14 (0.93-1.40) | 0.001 |
|  | **Grades 1-7** | 6,381 (24.1) | 1,452 | 15,216 | 9.5 (9.1-10.0) | 1 (reference) |  |
|  | **Grades 8-12** | 17,681 (66.8) | 4,403 | 40,073 | 11.0 (10.7-11.3) | 0.97 (0.90-1.04) |  |
|  | **College/Uni** | 1,915 (7.2) | 491 | 4,284 | 11.5 (10.5-12.5) | 1.19 (1.05-1.35) |  |
| **Marital status**  **(134 MV)** | **Currently married** | 13,990 (52.9) | 3,035 | 32,795 | 9.3 (8.9-9.6) | 1 (reference) | 0.039 |
|  | **Never married** | 9,272 (35.1) | 2,734 | 20,614 | 13.3 (12.8-13.8) | 1.10 (1.02-1.19) |  |
|  | **Previous married** | 3,181 (12.0) | 683 | 7,268 | 9.4 (8.7-10.1) | 1.06 (0.96-1.16) |  |
| **Employed**  **(69 MV)** | **No** | 6,510 (24.6) | 1,642 | 14,801 | 9.9 (9.4-10.4) | 1 (reference) | 0.330 |
|  | **Yes** | 19,998 (75.4) | 5,007 | 46,019 | 10.9 (10.6-11.2) | 1.04 (0.97-1.11) |  |
| **Sexual partners in last year**  **(3,690 MV)** | **None** | 5,202 (22.7) | 1,317 | 12,229 | 10.8 (10.2-11.4) | 1 (reference) | 0.379 |
|  | **One** | 16,199 (70.8) | 3,794 | 37,228 | 10.2 (9.9-10.5) | 0.96 (0.89-1.04) |  |
|  | **2-4** | 1,302 (5.7) | 386 | 3,022 | 12.8 (11.6-14.1) | 0.99 (0.88-1.12) |  |
|  | **5+** | 184 (0.8) | 69 | 398 | 17.3 (13.7-21.9) | 1.17 (0.90-1.51) |  |
| **Drug user**  **(161 MV)** | **No** | 25,726 (97.4) | 6,238 | 59,125 | 10.6 (10.3-10.8) | 1(reference) | 0.088 |
|  | **Yes** | 690 (2.6) | 209 | 1,485 | 14.0 (12.3-16.1) | 1.15 (0.98-1.34) |  |
| **Harmful alcohol use**  **(201 MV)** | **No** | 23,893 (90.6) | 5,769 | 54,648 | 10.6 (10.3-10.8) | 1 (reference) | 0.567 |
|  | **Yes** | 2,483 (9.4) | 659 | 5,801 | 11.4 (10.5-12.3) | 0.97 (0.89-1.07) |  |
| **Arm††**  **(0 MV)** | **A** | 9,305 (35.0) | 2,135 | 19,850 | 10.8 (10.3-11.2) | 1 (reference) | <0.001 |
|  | **B** | 7,844 (29.5) | 1,829 | 20,052 | 9.1 (8.7-9.5) | 0.88 (0.82-0.94) |  |
|  | **C** | 9,428 (35.5) | 2,521 | 21,054 | 12.0 (11.5-12.4) | 1.12 (1.05-1.20) |  |
| **Community****  **(0 MV)** | **Triplet 1, arm A** | 1,614 (6.1) | 376 | 3,706 | 10.1 (9.2-11.2) | 1 (reference) | <0.001 |
|  | **Triplet 1, arm B** | 1,882 (7.1) | 345 | 4,102 | 8.4 (7.6-9.3) | 0.89 (0.76-1.05) |  |
|  | **Triplet 1, arm C** | 2,052 (7.7) | 601 | 5,353 | 11.2 (10.4-12.2) | 1.08 (0.93-1.25) |  |
|  | **Triplet 2, arm A** | 2,644 (9.9) | 520 | 5,727 | 9.1 (8.3-9.9) | 0.73 (0.62-0.86) |  |
|  | **Triplet 2, arm B** | 2,283 (8.6) | 523 | 6,819 | 7.7 (7.0-8.4) | 0.58 (0.49-0.68) |  |
|  | **Triplet 2, arm C** | 2,630 (9.9) | 560 | 5,595 | 10.0 (9.2-10.9) | 0.90 (0.77-1.05) |  |
|  | **Triplet 3, arm A** | 2,347 (8.8) | 496 | 4,832 | 10.3 (9.4-11.2) | 0.90 (0.76-1.05) |  |
|  | **Triplet 3, arm B** | 1,779 (6.7) | 466 | 4,423 | 10.5 (9.6-11.5) | 1.04 (0.89-1.21) |  |
|  | **Triplet 3, arm C** | 2,659 (10.0) | 977 | 6,004 | 16.3 (15.3-17.3) | 1.51 (1.32-1.74) |  |
|  | **Triplet 4, arm A** | 2,700 (10.2) | 743 | 5,584 | 13.3 (12.4-14.3) | 1.17 (1.01-1.35) |  |
|  | **Triplet 4, arm B** | 1,900 (7.1) | 495 | 4,707 | 10.5 (9.6-11.5) | 0.90 (0.77-1.06) |  |
|  | **Triplet 4, arm C** | 2,087 (7.9) | 383 | 4,101 | 9.3 (8.4-10.3) | 0.81 (0.69-0.96) |  |
| **South Africa** | | | | | | | |
| **Gender¥ (98 MV)** | **Men** | 6,321 (31.1) | 1,835 | 15,316 | 12.0 (11.4-12.5) | 1 (reference) | <0.001 |
|  | **Women** | 14,002 (68.9) | 3,873 | 35,075 | 11.0 (10.7 – 11.4) | 0.86 (0.81-0.93) |  |
| **Age group¥ (102 MV)** | **18-19** | 1,954 (9.6) | 598 | 4,795 | 12.5 (11.5 - 13.5) | 0.93 (0.78-1.10) | <0.001 |
|  | **20-24** | 5,036 (24.8) | 1,652 | 12,005 | 13.8 (13.1 - 14.4) | 1 (reference) |  |
|  | **25-29** | 4,544 (22.4) | 1,456 | 11,004 | 13.2 (12.6 - 13.9) | 0.96 (0.88-1.04) |  |
|  | **30-34** | 3,721 (18.3) | 963 | 9,443 | 10.2 (9.6 - 10.9) | 0.73 (0.67-0.81) |  |
|  | **35-39** | 2,758 (13.6) | 606 | 7,117 | 8.5 (7.9 – 9.2) | 0.59 (0.53-0.66) |  |
|  | **40-44** | 2,306 (11.4) | 432 | 6,027 | 7.2 (6.5 - 7.9) | 0.52 (0.47-0.59) |  |
| **HIV status at entry (964 MV)** | **HIV-** | 15,163 (77.9) | 4,112 | 38,172 | 10.7 (10.4-11.1) | 1 (reference) | <0.001 |
|  | **HIV+ (SR-)** | 2,333 (12.0) | 835 | 5,443 | 15.2 (14.2-16.3) | 1.27 (1.17-1.38) |  |
|  | **HIV+ (SR+, ART-)** | 628 (3.2) | 214 | 1,490 | 14.4 (12.6-16.4) | 1.26 (1.09-1.46) |  |
|  | **HIV+ (SR+, ART+)** | 1,333 (6.9) | 355 | 3,167 | 11.2 (10.1-12.4) | 1.12 (1.00-1.27) |  |
| **Socio-economic**  **quintile**  **(396 MV)** | **1 (Poorest)** | 4,023 (20.1) | 1,453 | 9,365 | 15.5 (14.7-16.3) | 1 (reference) | <0.001 |
|  | **2** | 4,076 (20.4) | 1,251 | 9,502 | 13.2 (12.5-13.9) | 0.89 (0.82-0.97) |  |
|  | **3** | 4,262 (21.3) | 1,189 | 10,502 | 11.3 (10.7-12.0) | 0.75 (0.69-0.82) |  |
|  | **4** | 4,378 (21.9) | 1,040 | 11,561 | 9.0 (8.5-9.6) | 0.61 (0.56-0.67) |  |
|  | **5 (Richest)** | 3,286 (16.4) | 726 | 8,610 | 8.4 (7.8-9.1) | 0.67 (0.60-0.75) |  |
| **Education**  **(662 MV)** | **None** | 46 (0.2) | 12 | 122 | 9.8 (5.6-17.3) | 1.03 (0.53-2.00) | 0.102 |
|  | **Grades 1-7** | 1,547 (7.8) | 375 | 3,870 | 9.7 (8.8-10.7) | 1 (reference) |  |
|  | **Grades 8-12** | 34,856 (75.4) | 4,867 | 42,661 | 11.4 (11.1-11.7) | 1.01 (0.89-1.14) |  |
|  | **College/Uni** | 2,906 (6.3) | 295 | 2,324 | 12.7 (11.3-14.2) | 1.19 (1.00-1.19) |  |
| **Marital status**  **(386 MV)** | **Currently married** | 5,288 (26.4) | 1,355 | 13,305 | 10.2 (9.7-10.7) | 1 (reference) | 0.863 |
|  | **Never married** | 14,185 (70.8) | 4,176 | 34,863 | 12.0 (11.6-12.3) | 0.98 (0.91-1.06) |  |
|  | **Previous married** | 562 (2.8) | 133 | 1,394 | 9.5 (8.0-11.3) | 0.97 (0.79-1.18) |  |
| **Employed**  **(357 MV)** | **No** | 5,734 (28.6) | 1,436 | 14,215 | 10.1 (9.6-10.6) | 1 (reference) | 0.004 |
|  | **Yes** | 14,330 (71.4) | 4,234 | 35,401 | 12.0 (11.6-12.3) | 1.11 (1.03-1.19) |  |
| **Sexual partners in last year**  **(2,690 MV)** | **None** | 3,781 (21.3) | 1,068 | 9,233 | 11.6 (10.9-12.3) | 1 (reference) | 0.167 |
|  | **One** | 12,812 (72.3) | 3,605 | 31,710 | 11.4 (11.0-11.7) | 0.93 (0.86-1.00) |  |
|  | **2-4** | 1,004 (5.7) | 297 | 2,586 | 11.5 (10.2-12.9) | 0.89 (0.77-1.02) |  |
|  | **5+** | 134 (0.8) | 39 | 351 | 11.1 (8.1-15.2) | 0.83 (0.59-1.18) |  |
| **Drug user**  **(471 MV)** | **No** | 19,201(96.2) | 5,451 | 47,465 | 11.5 (11.2-11.8) | 1 (reference) | 0.189 |
|  | **Yes** | 749 (3.8) | 193 | 1,834 | 10.5 (9.1-12.1) | 0.89 (0.76-1.06) |  |
| **Harmful alcohol use**  **(781 MV)** | **No** | 17,802 (90.6) | 5,034 | 43,903 | 11.5 (11.2-11.8) | 1 (reference) | 0.008 |
|  | **Yes** | 1,838 (9.4) | 491 | 4,606 | 10.7 (9.8-11.6) | 0.86 (0.78-0.96) |  |
| **Arm††**  **(0 MV)** | **A** | 7,021 (34.4) | 2,036 | 16,761 | 12.1 (11.6 - 12.7) | 1 (reference) | 0.171 |
|  | **B** | 6,961 (34.1) | 1,944 | 18,170 | 10.7 (10.2 – 11.2) | 0.95 (0.89-1.03) |  |
|  | **C** | 6,439 (31.5) | 1,803 | 15,460 | 11.6 (11.1 – 12.1) | 1.02 (0.95-1.10) |  |
| **Community****  **(0MV)** | **Triplet 5, arm A** | 2,412 (11.8) | 823 | 5,574 | 14.7 (13.8-15.8) | 1 (reference) | <0.001 |
|  | **Triplet 5, arm B** | 2,510 (12.3) | 747 | 6,554 | 11.4 (10.6-12.2) | 0.87 (0.77-0.98) |  |
|  | **Triplet 5, arm C** | 2,176 (10.7) | 681 | 5,089 | 13.3 (12.4 (14.4) | 1.07 (0.94-1.22) |  |
|  | **Triplet 6, arm A** | 2,702 (13.2) | 878 | 6,133 | 14.3 (13.4 -15.3) | 1.12 (0.98-1.26) |  |
|  | **Triplet 6, arm B** | 2,402 (11.8) | 811 | 5,694 | 14.2 (13.2-15.2) | 1.17 (1.04-1.33) |  |
|  | **Triplet 6, arm C** | 2,599 (12.7) | 843 | 5,730 | 14.5 (13.6-15.6) | 1.12 (0.99-1.26) |  |
|  | **Triplet 7, arm A** | 1,907 (9.3) | 878 | 5,053 | 6.6 (6.0-7.2) | 0.56 (0.48-0.66) |  |
|  | **Triplet 7, arm B** | 2,049 (10.0) | 811 | 5,922 | 6.5 (5.9-7.2) | 0.52 (0.44-0.61) |  |
|  | **Triplet 7, arm C** | 1,664 (8.1) | 843 | 4,642 | 6.0 (5.3-6.7) | 0.54 (0.45-0.64) |  |

MV = Missing values

HIV- = Confirmed as HIV- in lab test

HIV+= Confirmed as HIV+ in lab test

SR- = Did not know or did not disclose their HIV+ status at the time of the PC visit

SR+ = Self-reported HIV+ status at time of PC visit

ART- = Did not report currently being on ART at time of PC visit

ART+ = Reported currently being on ART at time of PC visit

**p-values obtained from Poisson regression**

**[†Analyses are adjusted for gender, age (+age-gender interaction, except for age and gender estimates themselves) HIV status, education, marital status, employed, number of sexual partners in last year, drug use, alcohol use and community, unless specified ¥Does not include interaction between age and gender ††Only adjusted for triplet, not community **Not adjusted for arm]**
