# Supplementary material for: Migration and its impact on universal HIV testing and treatment in the HPTN 071 (PopART) study communities
Source: PLOS Glob Public Health. 2026 Jun 1;6(6):e0005357. doi: 10.1371/journal.pgph.0005357 (PMC13225650; doi:10.1371/journal.pgph.0005357)
Supplement: S2 File — (DOCX) [file pgph.0005357.s002.docx]

### Supplementary material S2 – Stratum-specific rate ratios for association between gender/age and out-migration

| **Exposure** | **Strata** | **Category** | **Zambia** | | **SA** | |
| --- | --- | --- | --- | --- | --- | --- |
|  |  |  | **Adjusted† Rate Ratio** | **p-value** | **Adjusted† Rate Ratio** | **p-value** |
| **Gender** | **18-19** | **Men** | 1 (baseline) | 0.085 | 1 (baseline) | 0.541 |
|  |  | **Women** | 1.32 (0.96-1.80) |  | 1.12 (0.77-1.63) |  |
|  | **20-24** | **Men** | 1 (baseline) | 0.236 | 1 (baseline) | 0.343 |
|  |  | **Women** | 0.94 (0.85-1.04) |  | 1.06 (0.94-1.21) |  |
|  | **25-29** | **Men** | 1 (baseline) | 0.001 | 1 (baseline) | 0.176 |
|  |  | **Women** | 0.81 (0.71-0.92) |  | 0.92 (0.81-1.04) |  |
|  | **30-34** | **Men** | 1 (baseline) | <0.001 | 1 (baseline) | 0.005 |
|  |  | **Women** | 0.63 (0.54-0.73) |  | 0.80 (0.69-0.93) |  |
|  | **35-39** | **Men** | 1 (baseline) | <0.001 | 1 (baseline) | <0.001 |
|  |  | **Women** | 0.59 (0.48-0.72) |  | 0.69 (0.58-0.83) |  |
|  | **40-44** | **Men** | 1 (baseline) | <0.001 | 1 (baseline) | <0.001 |
|  |  | **Women** | 0.65 (0.52-0.81) |  | 0.61 (0.51-0.73) |  |
| **Age group** | **Men** | **18-19** | 0.63 (0.47-0.83) | <0.001 | 0.89 (0.63-1.24) | 0.004 |
|  |  | **20-24** | 1 (baseline) |  | 1 (baseline) |  |
|  |  | **25-29** | 0.97 (0.85-1.10) |  | 1.06 (0.92-1.23) |  |
|  |  | **30-34** | 0.90 (0.77-1.05) |  | 0.90 (0.76-1.06) |  |
|  |  | **35-39** | 0.67 (0.55-0.80) |  | 0.80 (0.66-0.97) |  |
|  |  | **40-44** | 0.53 (0.43-0.64) |  | 0.77 (0.64-0.93) |  |
|  | **Women** | **18-19** | 0.88 (0.5-1.03) | <0.001 | 0.94 (0.76-1.15) | <0.001 |
|  |  | **20-24** | 1 (baseline) |  | 1 (baseline) |  |
|  |  | **25-29** | 0.84 (0.77-0.91) |  | 0.91 (0.83-1.01) |  |
|  |  | **30-34** | 0.60 (0.54-0.66) |  | 0.68 (0.61-0.75) |  |
|  |  | **35-39** | 0.42 (0.37-0.48) |  | 0.52 (0.46-0.59) |  |
|  |  | **40-44** | 0.36 (0.31-0.42) |  | 0.44 (0.39-0.51) |  |

[† Adjusted for HIV status, education, marital status, employed, number of sexual partners in last year, drug use, alcohol use and community]

HIV- = Confirmed as HIV- in lab test

HIV+= Confirmed as HIV+ in lab test

SR- = Did not know or did not disclose their HIV+ status at the time of the PC visit

SR+ = Self-reported HIV+ status at time of PC visit

ART- = Did not report currently being on ART at time of PC visit

ART+ = Reported currently being on ART at time of PC visit

p-values obtained from Poisson regression
