# Supplementary material for: Migration and its impact on universal HIV testing and treatment in the HPTN 071 (PopART) study communities
Source: PLOS Glob Public Health. 2026 Jun 1;6(6):e0005357. doi: 10.1371/journal.pgph.0005357 (PMC13225650; doi:10.1371/journal.pgph.0005357)
Supplement: S3 File — (DOCX) [file pgph.0005357.s003.docx]

### Supplementary material S3 – Detail on adjustment for primary trial outcome

There was some evidence of a difference in out-migration rates between trial arms in Zambia, although not in SA (shown in supplementary table S3). Also, plotting the community-level estimates of out-migration against the community-level estimates of HIV incidence showed a strong positive correlation (figure A, below). However, when plotted against the log ratio-residuals of observed vs expected incidence (i.e. incidence adjusted for age, gender, their interaction and baseline community HIV prevalence) no correlation appeared visible (figure B, below), suggesting that the association seen in figure A was confounded by age, sex and baseline prevalence. Hence why additionally adjusting for community level migration had no impact on the overall rate ratios.

Figure A - scatter plot and line of best fit between probability of out migration in one year and HIV incidence

Figure B - scatter plot and line of best fit between probability of out migration in one year and log of the ratio residuals
