# Supplementary material for: Migration and its impact on universal HIV testing and treatment in the HPTN 071 (PopART) study communities
Source: PLOS Glob Public Health. 2026 Jun 1;6(6):e0005357. doi: 10.1371/journal.pgph.0005357 (PMC13225650; doi:10.1371/journal.pgph.0005357)
Supplement: S4 File — (DOCX) [file pgph.0005357.s004.docx]

### Supplementary material S4 – 90-90 estimates before and after R3 stratified by migration status

|  | | | | **Zambia** | | | | | | **SA** | | | | | |
| --- | --- | --- | --- | --- | --- | --- | --- | --- | --- | --- | --- | --- | --- | --- | --- |
|  |  | |  | **Before** | | | **After** | | | **Before** | | | **After** | | |
|  |  | |  | Knows HIV status | On ART | ART coverage | Knows HIV status | On ART | ART coverage | Knows HIV status | On ART | ART coverage | Knows HIV status | On ART | ART coverage |
| All ages | | Men | Longer-term resident | 84.7% | 89.6% | 75.9% | 96.4% | 87.1% | 83.9% | 82.9% | 84.7% | 70.2% | 93.8% | 86.5% | 81.2% |
|  |  |  | Moved zone | 66.6% | 90.6% | 60.3% | 93.9% | 82.0% | 77.0% | 66.8% | 85.2% | 56.9% | 89.1% | 82.7% | 73.7% |
|  |  |  | Moved community | 49.1% | 91.4% | 44.9% | 92.5% | 76.7% | 70.9% | 54.1% | 75.3% | 40.8% | 85.7% | 76.3% | 65.3% |
|  |  | Women | Longer-term resident | 88.3% | 90.1% | 79.6% | 96.7% | 89.4% | 86.4% | 92.0% | 89.6% | 82.5% | 97.7% | 92.3% | 90.2% |
|  |  |  | Moved zone | 72.8% | 93.5% | 68.1% | 94.9% | 85.7% | 81.4% | 81.4% | 88.0% | 71.6% | 95.0% | 91.8% | 87.2% |
|  |  |  | Moved community | 58.7% | 94.0% | 55.1% | 94.0% | 81.5% | 76.6% | 71.3% | 87.7% | 62.5% | 93.1% | 88.7% | 82.6% |
| 18-29 | | Men | Longer-term resident | 67.0% | 76.9% | 51.6% | 94.5% | 70.5% | 66.6% | 59.8% | 67.2% | 40.2% | 87.5% | 66.5% | 58.2% |
|  |  |  | Moved zone | 39.9% | 82.8% | 33.0% | 91.6% | 64.8% | 59.3% | 36.2% | 72.2% | 26.2% | 80.8% | 68.0% | 55.0% |
|  |  |  | Moved community | 27.1% | 88.4% | 23.9% | 91.1% | 62.1% | 56.5% | 32.7% | 68.4% | 22.4% | 81.4% | 61.6% | 50.2% |
|  |  | Women | Longer-term resident | 79.6% | 83.3% | 66.3% | 96.1% | 81.6% | 78.4% | 82.4% | 82.2% | 67.7% | 95.8% | 85.5% | 81.9% |
|  |  |  | Moved zone | 59.7% | 92.3% | 55.1% | 94.1% | 77.9% | 73.2% | 71.3% | 83.4% | 59.4% | 93.5% | 87.8% | 82.1% |
|  |  |  | Moved community | 46.1% | 91.5% | 42.2% | 93.4% | 70.9% | 66.2% | 58.1% | 88.8% | 51.6% | 91.2% | 85.1% | 77.6% |
| 30+ | | Men | Longer-term resident | 87.4% | 91.0% | 79.5% | 96.7% | 89.3% | 86.3% | 86.5% | 86.6% | 74.9% | 94.8% | 89.2% | 84.6% |
|  |  |  | Moved zone | 72.2% | 91.5% | 66.1% | 94.4% | 84.9% | 80.1% | 75.6% | 87.0% | 65.8% | 91.5% | 86.1% | 78.8% |
|  |  |  | Moved community | 54.7% | 91.8% | 50.3% | 92.9% | 79.8% | 74.1% | 60.0% | 76.4% | 45.8% | 86.8% | 79.7% | 69.2% |
|  |  | Women | Longer-term resident | 91.3% | 92.2% | 84.2% | 96.9% | 91.8% | 88.9% | 95.0% | 91.6% | 87.0% | 98.3% | 94.3% | 92.7% |
|  |  |  | Moved zone | 80.8% | 94.1% | 76.0% | 95.4% | 90.0% | 85.9% | 86.7% | 89.9% | 77.9% | 95.7% | 93.6% | 89.6% |
|  |  |  | Moved community | 68.7% | 95.3% | 65.4% | 94.5% | 88.9% | 84.0% | 80.6% | 87.2% | 70.3% | 94.4% | 91.1% | 86.1% |

"Knows HIV status” is the percentage of PLHIV who are aware (and disclose) their HIV-positive status. “On ART” is the percentage of PLHIV who are on ART out of all those who are aware (and disclose) their HIV-positive status. “ART coverage” is the percentage of PLHIV who are on ART (i.e. the product of the two previous percentages)
