# Supplementary material for: Migration and its impact on universal HIV testing and treatment in the HPTN 071 (PopART) study communities
Source: PLOS Glob Public Health. 2026 Jun 1;6(6):e0005357. doi: 10.1371/journal.pgph.0005357 (PMC13225650; doi:10.1371/journal.pgph.0005357)
Supplement: S5 File — (DOCX) [file pgph.0005357.s005.docx]

## Supplementary material S5 – Members of the HPTN 071 (PopART) trial study team

We are grateful to all members of the HPTN 071 (PopART) Study Team, and to the study participants and their communities, for their contributions to the research.

The HPTN 071 (PopART) Study Team: Richard Hayes (London School of Hygiene & Tropical Medicine, UK), Sarah Fidler (Imperial College, UK), Nulda Beyers (University of Stellenbosch, South Africa), Helen Ayles (Zambart, Zambia; and London School of Hygiene & Tropical Medicine, UK), Peter Bock (University of Stellenbosch, South Africa), Wafaa El-Sadr (HIV Prevention Trials Network [HPTN] Leadership and Operations Centre, USA), Myron Cohen (HPTN Leadership and Operations Centre, USA), Susan Eshleman (HPTN Laboratory Centre, Johns Hopkins University, USA), Yaw Agyei (HPTN Laboratory Centre, Johns Hopkins University, USA), Estelle Piwowar-Manning (HPTN Laboratory Centre, Johns Hopkins University, USA), Virginia Bond (Zambart, Zambia; and London School of Hygiene & Tropical Medicine, UK), Graeme Hoddinott (University of Stellenbosch, South Africa), Deborah Donnell (HPTN Statistical and Data Management Centre [SDMC], USA), Sian Floyd (London School of Hygiene & Tropical Medicine, UK), Ethan Wilson (HPTN SDMC, USA), Lynda Emel (HPTN SDMC, USA), Heather Noble (HPTN SDMC, USA), David Macleod (London School of Hygiene & Tropical Medicine, UK), David Burns (NIAID, USA), Christophe Fraser (University of Oxford, UK), Anne Cori (Imperial College, UK), Nirupama Sista (HPTN Leadership and Operations Centre, USA), Sam Griffith (HPTN Leadership and Operations Centre, USA), Ayana Moore (HPTN Leadership and Operations Centre, USA), Tanette Headen (HPTN Leadership and Operations Centre, USA), Rhonda White (HPTN Leadership and Operations Centre, USA), Eric Miller (HPTN Leadership and Operations Centre, USA), James Hargreaves (London School of Hygiene & Tropical Medicine, UK), Katharina Hauck (Imperial College, UK), Ranjeeta Thomas (Imperial College, UK), Mohammed Limbada (Zambart, Zambia), Justin Bwalya (Zambart, Zambia), Michael Pickles (Imperial College, UK), Kalpana Sabapathy (London School of Hygiene & Tropical Medicine, UK), Ab Schaap (Zambart, Zambia; and London School of Hygiene & Tropical Medicine, UK), Rory Dunbar (University of Stellenbosch, South Africa), Kwame Shanaube (Zambart, Zambia), Blia Yang (University of Stellenbosch, South Africa), Musonda Simwinga (Zambart, Zambia), Peter Smith (Imperial College, UK), Sten Vermund (HPTN Executive Committee), Nomtha Mandla (University of Stellenbosch, South Africa), Nozizwe Makola (University of Stellenbosch, South Africa), Anneen van Deventer (University of Stellenbosch, South Africa), Anelet James (University of Stellenbosch, South Africa), Karen Jennings (City Health Department, Cape Town, South Africa), James Kruger (Department of Health, Western Cape), Mwelwa Phiri (Zambart, Zambia), Barry Kosloff (Zambart, Zambia; and London School of Hygiene & Tropical Medicine, UK), Lawrence Mwenge (Zambart, Zambia), Sarah Kanema (Zambart, Zambia), Rafael Sauter (University of Oxford, UK), William Probert (University of Oxford, UK), Ramya Kumar (Zambart, Zambia; and London School of Hygiene & Tropical Medicine, UK), Ephraim Sakala (Zambart, Zambia), Andrew Silumesi (Ministry of Health, Zambia), Tim Skalland (HPTN SDMC, USA), Krista Yuhas (HPTN SDMC, USA).
