# Supplementary material for: Migration and its impact on universal HIV testing and treatment in the HPTN 071 (PopART) study communities
Source: PLOS Glob Public Health. 2026 Jun 1;6(6):e0005357. doi: 10.1371/journal.pgph.0005357 (PMC13225650; doi:10.1371/journal.pgph.0005357)
Supplement: Attachment — Submitted filename: Reviewer responses - migration and UTT.docx [file pgph.0005357.s007.docx]

PLOS Global Public Health – reviewer responses

Dear Dr. Brault,

Many thanks to you and the reviewers for the points raised during the review process. These were all really useful and are important contributions to strengthening the research. I very much appreciate it. In this document I’ll attempt to address each one in turn.

Responses to reviewer 1

Line 158 – 162: The sentence could be improved. e.g. Using the PC data, the annual rate of out-migration was estimated using Poisson regression and converted to the risk of out-migration over a one-year period. Community estimates were age-standardised based on the population distribution enumerated in round 3 of the intervention delivery. Differences in out-migration levels between categories of HIV status were expressed as relative risks derived from the Poisson regression.

**Thank you for the suggestion, I have amended the paragraph to closer to your suggestion. (Lines 193-198 in marked up document)**

Line 164-166: The statistical test is to be clearly stated e.g. two-stage cluster-level analysis.

**Thank you. All reviewers suggested more detail could be added to the methods section so I have added detail to this section including as you have suggested clearly stating that this was a two stage cluster level analysis. Lines 207-210**

A statement confirming that the assumptions of the Poisson regression have been met is to be included.

**Statement added in line 247**

Line 175-183: The type of data for the outcome variable is to be stated.

**All four outcomes are binary, I have clarified this in line 230 by adding “binary” in brackets.**

Line 226: The term adjusted rate ratio may be abbreviated as aIRR in subsequent sections, and rate ratio may likewise be abbreviated as IRR.

**I have defined this in the first place each are mentioned (line 194 and line 296) and then subsequently used the abbreviation in the text (Although have used the full name in table headings)**

Ensure all percentage figures decimal points in the text are standardised e.g. 1 decimal point.
**I have added 1 decimal place to the percentages in lines 329-342 to fix this**

Line 276: figure 3 (cap F).
Table 1, Table 3. Supplementary Table 3: Since the symbol (%) is already indicated at the top of the table, it can be omitted from the individual values.
Table 2:- association : Cap A.
Table 2: On the first row, column 4-7, 95% CI is to be stated.

**Thank you for these four observations – I have edited these all as suggested**

The additional statistical analyses info in the supplementary file could be moved to the Statistical Methods section.

**Thanks very much for this suggestion, this was reported by all reviewers and as such I have moved the content from supplementary material S2 into the statistical methods. I had originally prioritised brevity, but can appreciate that this led to some important information being omitted from the original main manuscript**.

For Table 2 footnote [* Adjusted for gender, age (and their interaction) and community † Adjusted for gender, age (and their interaction) community, socio-economic status, marital status, education, employment status, alcohol use, drug use, sexual partners in last year], the footnotes should follow those indicated in the supplementary files where applicable, and should specify the actual interaction variable(s). The same applies to Table 4. For [† Adjusted for gender, age (and their interaction) community], a comma should be placed after the closing bracket.

**I struggled slightly to parse this comment but just to try and summarise how I think I understood it**

- **I need to add square brackets around the footnotes that specify what is adjusted for**
  - **Done**
- **The footnotes should follow those indicated in the supplementary material**
  - **I understood this to mean that the things I say in the footnotes that I adjust for, matches the text that is in the statistical methods (originally in supplementary, now in main text). I think that it does but…**
- **And should specify the actual interaction variables**
  - **I’m guessing here that “their interaction” wasn’t specific enough, so I have changed that so it now says age-sex interaction. I hope this edit satisfies this and the above point**
- **A comma should be placed after the closing bracket**
  - **Have done so (I wasn’t aware of this convention)**

Supplementary material 3 table footnote: For [††Only adjusted for triplet, not community] & Line 92-93: Specify the categories (coding) for the communities in the statistical methods section.

**Thank you I have added the clarification to lines 99-100 of the methods:**

**“triplets” (triplets 1-4 in Zambia and triplets 5-7 in South Africa)”**

In the tables where statistical tests were performed, the name of the test is to be indicated in the table footnote.

**Thank you. I have added these to the relevant table footnotes**

For the statistical tests mentioned in the Statistical Methods section, these should also be clearly identified in the Results section, including in the relevant tables and figures. **I have clarified that the p-values were obtained from Poisson regression (table 2, supplementary table 1, supplementary table 2), logistic regression (table 4)**

The name of the statistical software, including the publisher, version, and the level of statistical significance adopted in the analyses, are to be stated.

**I have added “Stata version 18 (Statacorp) was used to perform all analyses.” to line 249**

**I am reluctant to list a level of statistical significance to the methods section. While I appreciate many people would deem a level of statistical significance of 5% as being important, there is a growing consensus among statisticians (including me!) that to dichotomise results at a specific p-value is of little benefit. So I have not added this.**

The information on missing values and their handling in the analyses is be described in the statistical methods section.

**This has been added at the end of the statistical methods section at line 248 “In all methods, complete case analyses were performed so no imputation of missing data was done.”**

Responses to reviewer 2

1) I would ask that if possible the authors add some clarification in the discussion about the type of migration examined in this study (ie whether short-term or long-term migration) - these results seem to me more applicable to long-term migration, although some study sites are well-known for seasonal migration (e.g. between Eastern and Western Cape in South Africa).

**This is a good point and I agree with the reviewer that this was unclear. The reviewer is correct that the data collected more closely relates to long-term migration rather than shorter-term/seasonal migration. I have added the following sentence at lines 416-418 to clarify this:**

**“These results are primarily applicable to long-term migration (rather than short-term, seasonal migration) as participants who temporarily relocated should have been retained in the cohort.”**

2) Can the authors just contextualise their discussion that the results are for adults aged 18-44 (plus any additional limitations that would prevent individuals from being seen in the PC/CHIP data).

**Thank you, I agree this is important. In the discussion I have added the age group 18-44 for the PC data analysis and 18+ for the CHiP analysis.**

**I have listed what I believe are the two largest limitations on the included participants. For the PC data the big limitation on the selection of the sample was exclusion of those who reported they were likely to leave within the three years of the study, and that the control arm didn’t have CHiP data at all.**

**While it is true that the sample overrepresents women, gender was accounted for in any measure of association between HIV and migration. While It is possible that the association between HIV and migration is different in those included in the sample and those not included beyond characteristics that we measured (i.e. people who are “hard to reach” in some difficult to quantify way) and that it is possible that the association between HIV and migration differs in this group, I’m not sure which direction I might expect the bias to occur though and so any statement of this as a limitation I felt might be too broad to be of use. So I have not added anything more to the limitations section on this.**

3) Can the authors confirm if the analysis is a complete-case analysis (or how missing data was dealt with).

**This has been added at the end of the statistical methods section at line 248 “In all methods, complete case analyses were performed so no imputation of missing data was done.”**

Responses to reviewer 3

Title: As this is not a journal dedicated specifically to HIV/AIDS, I suggest that the authors indicate that the universal testing and treatment approach is for HIV.
**This is a good point and I have added it to the title “Migration and its impact on universal HIV testing and treatment in the HPTN 071 (PopART) study communities”**

Abstract: Similarly to the title, acronyms related to HIV/AIDS are not trivial for readers of this review. I suggest that authors pay attention to the acronyms used in the abstract and throughout the text for an initial explanation (e.g., HIV, AIDS, UN, UNAIDS, HPTN, etc.).

AND

Introduction: As in the abstract, I suggest that authors look into explaining the acronyms.

**Thank you, I have added these all at first mention now (Although I have left HPTN in the title and abstract as it is the name of the trial, but defined it in the introduction).**

I suggest that the authors provide more detail on the HIV-related outcomes and the migration variable as the main independent variable in the analysis in the methods section of the abstract.

AND

In the results section, the authors present the associations without mentioning the measures of association and their respective confidence intervals.

**I have added this detail into a reviewed abstract (lines 39-41 and 49-52)) and I agree that this is important information. This has pushed the word count of the abstract up to 348, which I am hoping the editors will forgive me for….**

The authors present the result as follows: "out-migration was higher among HIV-positive individuals...". This interpretation may mislead readers into thinking that migration is the dependent variable, and HIV is the independent variable. I suggest inverting this interpretation, since the outcome is HIV (e.g., individuals who migrated out were more likely to be unaware or not disclose their HIV-positive status...).

AND

Between lines 220 and 224, the situation is the same as mentioned in the abstract; I suggest inverting the interpretation to better clarify migration as an independent variable.

AND

In the first paragraph of the discussion, I also suggest that authors consider the inversion of interpretation.

**Thank you very much for this important comment, it was in fact something we thought about carefully when preparing the manuscript and did consider what you have suggested. I agree it seems more natural to think of the health outcome of HIV as the dependent variable and migration as the independent variable. However, in the case of how we measured out-migration (from the PC data) the category of HIV status was measured at baseline, and any migration out of the community occurred subsequent to that. So a participant’s HIV status definitely preceded whether they migrated or not. For this reason we chose to report migration status as the dependent and HIV as the independent.**

**For the in-migration analysis the migration status preceded the collection of the HIV status (although the timing of migration status and HIV status is unclear given the data are cross-sectional) and as such it made more sense to treat HIV status as the dependent variable. This is why for the two data sets we report the results in slightly different ways.**

**However, in considering this point I have noticed throughout the paper I have used the phrase “HIV outcomes” to group together the HIV-related indicators of HIV-positive status, knowledge of status and ART use, which I think could cause confusion as these “outcomes” are sometimes used as the dependent variable. So I have replaced this term (“HIV outcomes”) throughout with a more neutral term (HIV-related indicators). I do continue to describe as outcomes when describing in-migration specifically.**

Between lines 79 and 86, the difference between objective 1 and objective 3 is unclear. Wouldn't objective 1 be included in objective 3?

**I think it is important to keep objective 1 and 3 separate as objective 1 relates to migration *out* of the trial communities (and as described above HIV status precedes migration) whereas objective 3 relates to migration *into or within* the trial communities. So while we are indeed looking at associations between the same things (HIV indicators and people movement) the sources of data and the nature of the people movement may be different and combining these objectives may cause some confusion. I wonder if some of the confusion between objective 1 and objective 3 came about because of my slack use of the word outcomes, which I have now amended….**

Methods: The definition of variables and statistical analysis described in the methods are unclear. Reading the supplementary information S1 and S2, the authors should consider including them in the text of the article, as they are essential for understanding the strategies used and for the strength of the article.

**I have added the detailed statistical methodology and the methods for categorising in-migrants into the main body of the text as requested.**

Results: I suggest that the authors describe sociodemographic characteristics so that readers can better understand the study population.

**Thank you, I agree with this suggestion and this has now been added in lines 264-267**

Discussion: I missed the authors discussing possible regional differences in the results presented. I also suggest a discussion of the results with other results in the literature.

**I have now added a paragraph more fully describing the results in context of other literature (lines 420-430) and the differences/similarities between the two countries in lines 445-455.**

Figure 1: A footnote explaining what T1, T2, T3, etc. is missing.

**I have added this in (Line 282)**

Figure 3: Would it be possible to increase the size of the graphs? It is not easy to compare them at their current size.

**Yes I have re-generated all figures in TIF format and enlarged. I hope these are more suitable. I have passed them through the PLOS NAAS tool to check if they meet criteria and seem to be OK**

Responses to editor comments and journal requirements

All three of the reviewers request clarifications concerning methods, and I agree with the suggestions to shift some of the supplemental methods information into the main text.

**Thank you for this, I agree and have added in supplementary materials S1 and S2 into the main body of the paper.**

I also agree with the reviewers' requests for an expanded discussion that clarifies and discusses the implications of the types of migration examined in the manuscript. Better linkage to the literature overall is needed in the discussion, and I would recommend additional attention to regional differences if any, and implications/recommendations for HIV prevention and care based on the findings.

**Thanks, yes I have added two paragraphs to the discussion that I hope has addressed the reviewers concerns with respect to these points.**

1. Please include a complete copy of PLOS’ questionnaire on inclusivity in global research in your revised manuscript. Our policy for research in this area aims to improve transparency in the reporting of research performed outside of researchers’ own country or community. The policy applies to researchers who have travelled to a different country to conduct research, research with Indigenous populations or their lands, and research on cultural artefacts. The questionnaire can also be requested at the journal’s discretion for any other submissions, even if these conditions are not met.  Please find more information on the policy and a link to download a blank copy of the questionnaire here: [https://journals.plos.org/globalpublichealth/s/best-practices-in-research-reporting](https://url.uk.m.mimecastprotect.com/s/mb80CqZ14tKkpVLFZf3CEllXN?domain=journals.plos.org). Please upload a completed version of your questionnaire as Supporting Information when you resubmit your manuscript.

**This has been completed and uploaded along with the resubmission**

2. Please provide a detailed online Financial Disclosure statement. This is published with the article. It must therefore be completed in full sentences and contain the exact wording you wish to be published.

a) State the initials, alongside each funding source, of each author to receive each grant. For example: “This work was supported by the National Institutes of Health (####### to AM; ###### to CJ) and the National Science Foundation (###### to AM).”
b) State what role the funders took in the study. If the funders had no role in your study, please state: “The funders had no role in study design, data collection and analysis, decision to publish, or preparation of the manuscript.”

For more information, please go to our submission guidelines:
[https://journals.plos.org/globalpublichealth/s/submission-guidelines#loc-financial-disclosure-statement](https://url.uk.m.mimecastprotect.com/s/1iRSCr914SPn5WwHzhpC4G0b7?domain=journals.plos.org)

**Thanks for this. In reviewing and double checking what I had provided I consulted with our project administrator. She has advised me that the NIH (from mid-2023) had requested that we no longer add the grant numbers. So as a result of this I have actually removed them from the acknowledgements of the manuscript. The financial disclosure paragraph is as follows:**

**“This work was supported by the National Institute of Allergy and Infectious Diseases, the US President’s Emergency Plan for AIDS Relief, the International Initiative for Impact Evaluation, the Bill and Melinda Gates Foundation, the National Institute on Drug Abuse, and the National Institute of Mental Health The funders had no role in study design, data collection and analysis, decision to publish, or preparation of the manuscript.”**

3. Please ensure that the funders and grant numbers match between the Financial Disclosure field and the Funding Information tab in your submission form. Note that the funders must be provided in the same order in both places as well.

**As mentioned above the funder has requested that we do not publish the specific grant numbers in the manuscript, but I have added them in the funding information tab**

4. Please update your online Competing Interests statement. If you have no competing interests to declare, please state: “The authors have declared that no competing interests exist.”

**Done**

5. In the online submission form, you indicated that “Requests for the data may be sent to [HPTN-Data-Access@scharp.org](mailto:HPTN-Data-Access@scharp.org).”.

All PLOS journals now require all data underlying the findings described in their manuscript to be freely available to other researchers, either
a) In a public repository,
b) Within the manuscript itself, or
c) Uploaded as supplementary information.

This policy applies to all data except where public deposition would breach compliance with the protocol approved by your research ethics board. If your data cannot be made publicly available for ethical or legal reasons (e.g., public availability would compromise patient privacy), please explain your reasons by return email and your exemption request will be escalated to the editor for approval. Your exemption request will be handled independently and will not hold up the peer review process, but will need to be resolved should your manuscript be accepted for publication. One of the Editorial team will then be in touch if there are any issues.

**Thanks for this, the datasets are freely available to download from the Harvard Dataverse:** [**https://dataverse.harvard.edu/dataverse/HPTN-071**](https://dataverse.harvard.edu/dataverse/HPTN-071) **. I have updated the information on the online submission to reflect this.**

6. Please provide separate main figure files in .tif or .eps format only and ensure that all files are under our size limit of 10MB.

For more information about how to convert your figure files please see our guidelines: [https://journals.plos.org/globalpublichealth/s/figures](https://url.uk.m.mimecastprotect.com/s/BDUgCvZ14tpLDROizimCQuhXv?domain=journals.plos.org)

**This has been done now and checked with the NAAS tool.**

7. If the reviewer comments include a recommendation to cite specific previously published works, please review and evaluate these publications to determine whether they are relevant and should be cited. There is no requirement to cite these works unless the editor has indicated otherwise.

**N/A**

8. Please review your reference list to ensure that it is complete and correct. If you have cited papers that have been retracted, please include the rationale for doing so in the manuscript text, or remove these references and replace them with relevant current references. Any changes to the reference list should be mentioned in the rebuttal letter that accompanies your revised manuscript. If you need to cite a retracted article, indicate the article’s retracted status in the References list and also include a citation and full reference for the retraction notice.

**Complete**
